# Diagnostic value of CSF CXCL8 combined with total protein for neurosyphilis: a logistic regression and ROC analysis

**DOI:** 10.3389/fimmu.2026.1806528

**Published:** 2026-06-03

**Authors:** Yan Zhang, Yujiao Jin, Yuan Liu, Lizhi Xue, Kailong Gu, Wei Du, Shourong Liu, Aifang Xu

**Affiliations:** 1 Department of Clinical Laboratory, Affiliated Hangzhou Xixi Hospital, Zhejiang Chinese Medical University, Hangzhou, China; 2 Department of Infection, Affiliated Hangzhou Xixi Hospital, Zhejiang Chinese Medical University, Hangzhou, China

**Keywords:** cerebrospinal fluid, CXCL1, CXCL5, CXCL8, neurosyphilis

## Abstract

**Background:**

Neurosyphilis has resurged globally, presenting a significant public health threat, yet challenges in early diagnosis persist. This study aims to evaluate the diagnostic value of C-X-C motif chemokine ligand 1 (CXCL1), C-X-C motif chemokine ligand 5(CXCL5), and C-X-C motif chemokine ligand 8 (CXCL8) in cerebrospinal fluid (CSF) for neurosyphilis.

**Methods:**

A total of 126 patients were included in this study, comprising 44 syphilis patients and 82 diagnosed with neurosyphilis (28 asymptomatic and 54 symptomatic). We assessed CSF chemokines, CSF parameters, and lymphocyte subpopulations. Univariate and multivariate logistic regression analyses were conducted to identify predictors of neurosyphilis. Receiver operating characteristic (ROC) curve analysis was employed to evaluate the diagnostic value of individual and combined biomarkers for this condition.

**Results:**

CSF CXCL1 and CSF CXCL8 levels were significantly elevated in the neurosyphilis group compared to the syphilis group (P < 0.05). Additionally, CSF white cell count (CSF WBC) and CSF total protein (CSF TP) levels were increased, while CD4 levels were decreased. Binary logistic regression analysis identified CSF CXCL8 and CSF TP as independent predictors of neurosyphilis. ROC curve analysis revealed areas under the curve (AUC) for CSF CXCL8 and CSF TP distinguishing neurosyphilis from syphilis of 0.766 and 0.830, respectively. The combined assessment of CSF CXCL8 and CSF TP further improved diagnostic accuracy, with an AUC of 0.865.

**Conclusions:**

The levels of CSF CXCL1 and CSF CXCL8 are valuable for diagnosing neurosyphilis and assessing treatment efficacy. The combined detection of CSF CXCL8 and CSF TP enhances diagnostic precision.

## 1 Introduction

Neurosyphilis, caused by the invasion of the central nervous system by *Treponema pallidum*, represents the most severe complication of syphilis. With the resurgence of syphilis globally, the incidence of neurosyphilis has also increased (1, 2). The clinical manifestations of neurosyphilis are diverse, and patients often overlook early signs of syphilis or asymptomatic neurological involvement, leading to delays in diagnosis and treatment. Consequently, neurosyphilis remains a significant public health concern (1, 3, 4).

Currently, laboratory diagnosis of neurosyphilis relies primarily on cerebrospinal fluid examinations, including both treponemal and non-treponemal tests, as well as assays for CSF white blood cell (CSF WBC) and CSF total protein (CSF TP) levels. Venereal Disease Research Laboratory (VDRL) titer of the CSF is considered the traditional “gold standard” (5–7), demonstrating high specificity but low sensitivity. No single test currently exists that can definitively diagnose or exclude neurosyphilis (1, 8). Therefore, identifying a sensitive and easily operable diagnostic method is essential.

Chemokines, a class of cytokines with chemotactic properties, play crucial roles in maintaining homeostasis, mediating acute inflammation, and regulating adaptive immune responses by coordinating the migration and localization of immune cells at the organ level (9). Research suggests that chemokines are biological markers in various central nervous system tumors and diseases, and they play a key role in the progression of neurosyphilis. Assessing chemokine levels may facilitate early diagnosis and treatment of this condition (10–14).

In our study, we investigated the levels of C-X-C motif chemokine ligand 1 (CXCL1), C-X-C motif chemokine ligand 5(CXCL5), and C-X-C motif chemokine ligand 8 (CXCL8) in the CSF of patients with neurosyphilis. Logistic regression analysis was employed to identify predictive factors for neurosyphilis. Additionally, receiver operating characteristic (ROC) curve analysis was conducted to evaluate the diagnostic value of chemokines and their combinations as biomarkers. Our results indicate that CSF CXCL8 has potential diagnostic value in neurosyphilis. The application of these biomarkers is expected to enhance proactive prevention and management strategies for neurosyphilis.

## 2 Materials and methods

### 2.1 Study design and patient selection

This prospective study was conducted within an institution from January 2023 to August 2025. The study participants included patients undergoing lumbar puncture screening for neurosyphilis and those receiving treatment for neurosyphilis during the follow-up period. A total of 126 participants were recruited, comprising 44 syphilis patients and 82 patients with neurosyphilis (including 28 asymptomatic and 54 symptomatic cases). Additionally, 15 neurosyphilis patients were followed up three months post-treatment to compare chemokine levels before and after therapy.

The inclusion criteria were based on the 2015 UK National Syphilis Management Guidelines (15) and the 2020 Chinese Guidelines for Sexually Transmitted Infections (16). The exclusion criteria included:1. Patients under 18 years of age. 2. Positive human immunodeficiency virus (HIV) antibody test. 3. Traumatic CSF collection. 4. History of other central nervous system diseases. All participants underwent HIV serological testing prior to enrollment. To avoid the potential confounding effects of HIV-associated immunodeficiency on inflammatory biomarkers, individuals with confirmed HIV infection were excluded from this study.

**Figure 1:**
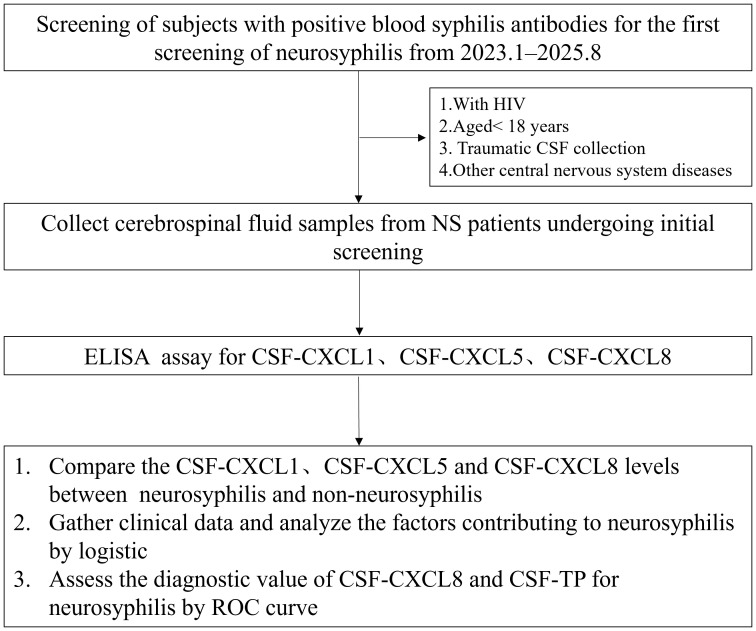
Flowchart of all participants in the study.

This study was approved by the Medical Ethics Committee of the Affiliated Hangzhou Xixi Hospital, Zhejiang Chinese Medical University (Ethical approval number: 2024-027). The study flowchart is illustrated in **Figure 1**.

### 2.2 Data collection

We collected demographic and clinical data through the hospital’s Laboratory Information System. The collected data comprised age, sex, lymphocyte subpopulations, rapid plasma regain (RPR) test results, and treponema pallidum particle agglutination assay (TPPA) results. Additionally, CSF parameters were recorded, including CSF WBC, CSF red blood cell (CSF RBC), CSF glucose (CSF GLU), CSF TP, CSF adenosine deaminase (CSF ADA), CSF lactate dehydrogenase (CSF LDH), CSF RPR, and CSF TPPA. The collection of CSF samples was a routine diagnostic procedure, with samples stored at −80 °C for subsequent analysis.

CSF samples were analyzed using the following R&D Systems ELISA kits: CXCL1 (Human CXCL1/GRO alpha Quantikine ELISA Kit), CXCL5 (Human CXCL5/ENA-78 Quantikine ELISA Kit), and CXCL8 (Human IL8/CXCL8 Quantikine ELISA Kit). All cytokines were measured according to the manufacturer’s instructions, with absorbance assessed at a wavelength of 450 nm. The concentrations of cytokines were calculated based on standard curves.

### 2.3 Statistical analysis

Statistical analyses were conducted using IBM SPSS Statistics (Version 25.0) and GraphPad Prism version 8.0.2 (GraphPad Software, San Diego, CA, USA). Categorical variables are presented as proportions, while continuous variables are reported as medians with interquartile ranges (IQR). To compare differences between groups, the Mann-Whitney U test was applied for continuous variables, and the χ² test was utilized for categorical variables. A subgroup analysis was conducted among patients diagnosed with neurosyphilis to compare chemokine levels between asymptomatic and symptomatic individuals. For those neurosyphilis patients who were followed up post-treatment, changes in chemokine levels in cerebrospinal fluid were analyzed using the Wilcoxon signed-rank test. Due to the limited sample size, analyses regarding asymptomatic neurosyphilis and comparisons of pre- and post-treatment chemokine levels are considered exploratory. A binary logistic regression model was constructed to diagnose neurosyphilis, and the diagnostic performance of these indicators was assessed using receiver operating characteristic (ROC) curves. CSF RPR showed complete separation between the study groups, meaning that the variable perfectly distinguished the outcome categories in the logistic regression model. Under this condition, the maximum likelihood estimation failed to converge, resulting in unstable or non-estimable regression coefficients and inflated standard errors. Consequently, reliable ROC model estimation could not be achieved for CSF RPR, and it was therefore excluded from the ROC analysis. A p-value of <0.05 was deemed statistically significant.

## 3 Results

### 3.1 Characteristics of the study participants

A total of 126 participants were included in this study, consisting of 69 males and 57 females. **Table 1** summarizes the basic clinical information for both the syphilis and neurosyphilis groups. The mean age of the neurosyphilis group was higher than that of the syphilis group, with no significant differences in gender distribution between the two groups. Notably, 1/Serum RPR titer, CSF RPR (+), CSF TPPA (+), CSF WBC, CSF TP, and CSF ADA levels were significantly elevated in the neurosyphilis group compared to the syphilis group. However, no statistically significant differences were observed between the groups for TPPA (+), CSF RBC, CSF GLU, and CSF LDH (*P* > 0.05). Furthermore, levels of CD4+ T cells and B cells were significantly lower in patients with neurosyphilis than in those with syphilis (*P* < 0.05), while no significant differences were noted in NK cell levels and CD8+ T cells between the two groups.

**Table 1:** Baseline data of syphilis patients and neurosyphilis patients.

Variables	Total (n = 126)	Syphilis (n = 44)	Neurosyphilis (n = 82)	*P*
Age, years	48.61 ± 15.05	42.86 ± 16.75	51.70 ± 13.16	0.003
Male, n (%)	69 (54.76)	19 (43.18)	50 (60.98)	0.056
CD3+ T cells (/μL)	1109.50 (837.25, 1387.25)	1144.50 (1004.50, 1496.00)	1094.50 (794.00, 1285.00)	0.085
CD4+ T cells (/μL)	573.50 (441.50, 783.00)	726.50 (551.50, 978.50)	543.50 (416.75, 687.50)	0.006
CD8+ T cells (/μL)	417.50 (304.75, 521.00)	422.50 (333.00, 522.25)	401.50 (298.50, 518.75)	0.432
B cells (/μL)	195.50 (126.00, 273.00)	260.50 (181.50, 290.75)	161.50 (104.75, 266.25)	0.003
NK cells (/μL)	235.00 (147.75, 317.50)	221.00 (161.00, 377.75)	240.00 (133.00, 316.00)	0.809
1/Serum RPR titer	8.00 (2.00, 16.00)	6.00 (2.00, 16.00)	12.00 (4.00, 28.00)	0.021
TPPA (+), n (%)	123 (97.62)	42 (95.45)	81 (98.78)	0.579
CSF RPR (+), n (%)	32 (25.40)	0 (0.00)	32 (39.02)	<.001
CSF TPPA (+), n (%)	82 (65.08)	12 (27.27)	70 (85.37)	<.001
CSF WBC (cells/uL)	2.00 (1.00, 4.00)	2.00 (1.00, 3.25)	2.00 (2.00, 5.00)	0.020
CSF RBC (cells/uL)	1.00 (0.00, 4.00)	1.50 (0.00, 4.25)	1.00 (0.00, 4.00)	0.592
CSF GLU (mmol/L)	3.30 (3.00, 3.77)	3.30 (3.00, 3.82)	3.30 (3.00, 3.70)	0.760
CSF TP (mg/L)	447.50 (333.25, 609.25)	334.50 (282.25, 427.50)	543.00 (405.00, 653.00)	<.001
CSF ADA (U/L)	0.40 (0.30, 0.60)	0.30 (0.20, 0.30)	0.50 (0.30, 0.70)	<.001
CSF LDH (U/L)	14.00 (7.00, 19.00)	11.00 (7.75, 15.25)	15.00 (7.00, 19.00)	0.105

CSF, cerebrospinal fluid; RPR, rapid plasma regain test; TPPA, treponema pallidum particle agglutination assay; WBC, white blood cell; RBC, red blood cell; GLU, glucose; TP, total protein; ADA, adenosine deaminase; LDH, lactate dehydrogenase.

### 3.2 Assessment of CSF CXCL1, CXCL5, and CXCL8 levels of neurosyphilis and syphilis patients

**Figure 2:**
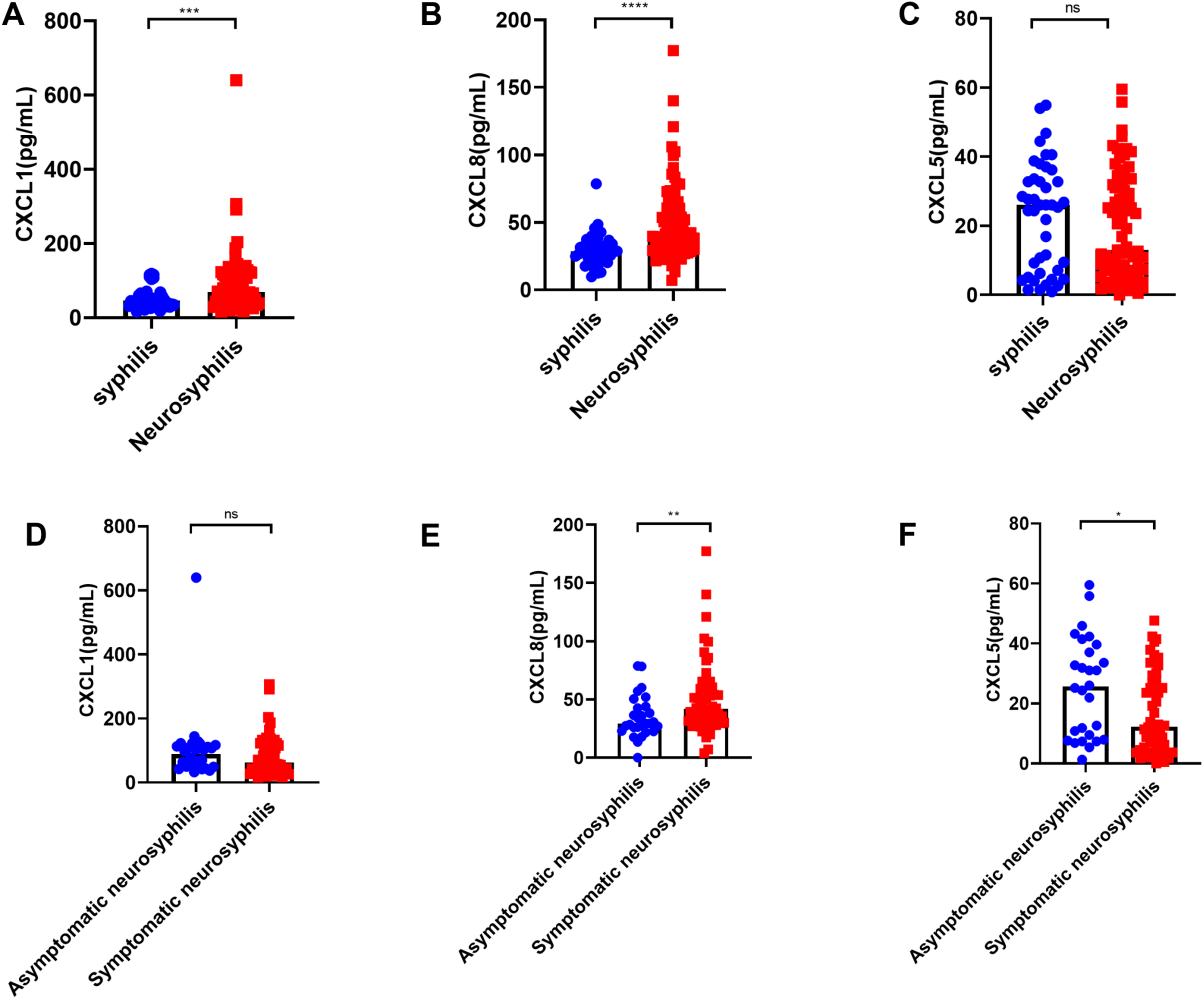
Chemokine levels. **(A)** Levels of CSF CXCL1 of patients with syphilis and neurosyphilis. **(B)** Levels of CSF CXCL8 of patients with syphilis and neurosyphilis. **(C)** Levels of CSF CXCL5 of patients with syphilis and neurosyphilis. **(D)** Levels of CSF CXCL1 of patients with symptomatic and asymptomatic neurosyphilis. **(E)** Levels of CSF CXCL8 of patients with symptomatic and asymptomatic neurosyphilis. **(F)** Levels of CSF CXCL5 of patients with symptomatic and asymptomatic neurosyphilis. Data are presented as medians with IQR. Statistical analysis was performed using Mann-Whitney U test. Neurosyphilis (n=82), syphilis (n=44). *p < 0.05, **p < 0.01, ***p < 0.001, ****p < 0.001.

Levels of CSF CXCL1 and CXCL8 were significantly higher in the neurosyphilis group compared to the syphilis group, while no significant difference was observed in CXCL5 levels between the two groups (**Figures 2A-C**). A subgroup analysis among patients diagnosed with neurosyphilis was performed to compare chemokine levels between asymptomatic and symptomatic individuals. Notably, the CSF CXCL8 levels in the symptomatic neurosyphilis patients were significantly higher than those in asymptomatic neurosyphilis patients, whereas CXCL5 levels showed the opposite trend. The levels of CSF CXCL1 did not differ significantly between the two groups (**Figures 2D-F**).

### 3.3 Diagnostic value of CSF chemokines and parameters in patients with neurosyphilis

Significant variables identified through univariate analysis were categorized into clinical indicators, serological markers, CSF inflammatory markers, and chemokine-related indicators. Initial logistic regression analyses were conducted within each category to determine representative predictive factors. Subsequently, these selected variables were incorporated into a final multivariate logistic regression model to mitigate issues of multicollinearity and overfitting.

In the final multivariate logistic regression model (which excluded the RPR parameter from the cerebrospinal fluid), both CSF TP (OR = 1.008, 95% confidence interval [CI]: 1.004–1.012, P < 0.001) and CSF CXCL8 (OR = 1.056, 95% CI: 1.017–1.095, P = 0.004) were independently associated with the occurrence of neurosyphilis (**Table 2**). Based on the expression levels of these indicators, a predictive model was established to calculate the probability score for each patient, with the formula: Probability Score = -4.856 + 0.054 (CSF CXCL8) + 0.008 (CSF TP).

**Table 2:** Logistic regression analysis of risk factors in patients with neurosyphilis.

Variable	B	Standard error	Waldχ2	P-value	Exp(B)	(95%CL)
CSF CXCL8 (pg/mL)	0.054	0.019	8.283	0.004	1.056	(1.017~1.095)
CSF TP (g/L)	0.008	0.002	16.862	0.000	1.008	(1.004~1.012)
Constant	-4.856	1.049	21.450	0.000	0.008	

### 3.4 Performance of CSF CXCL8 and CSF TP in diagnosing neurosyphilis

**Figure 3:**
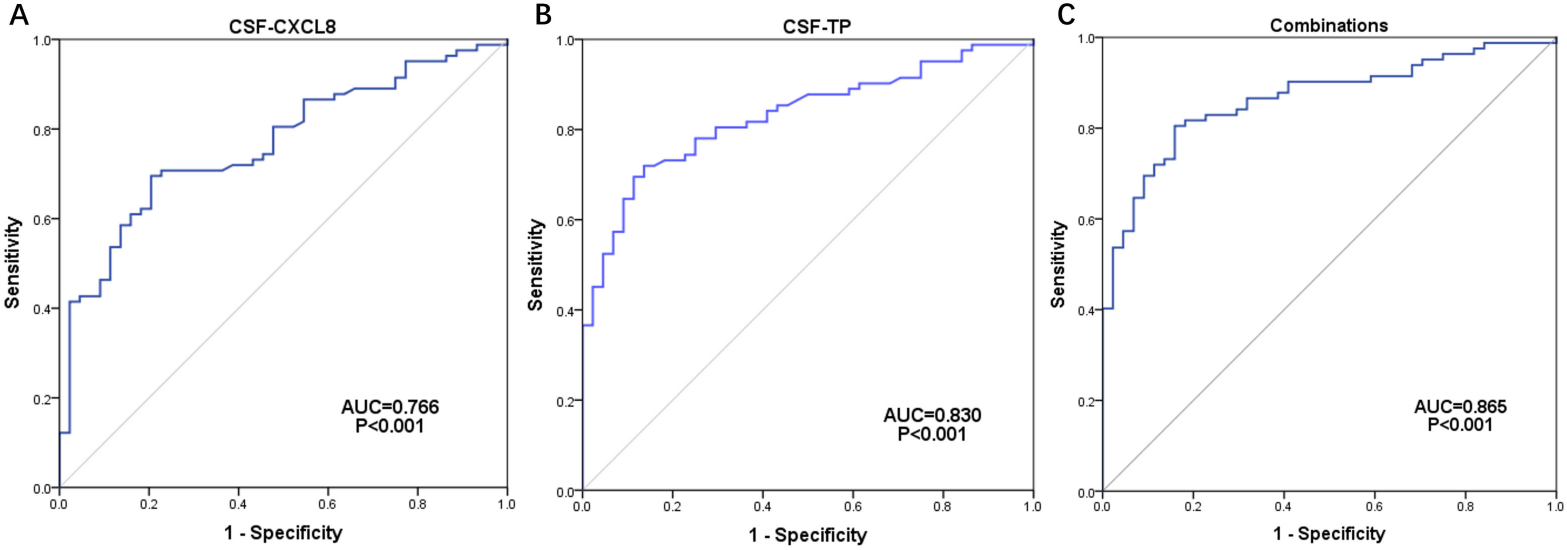
Diagnostic value of CSF CXCL8 and CSF TP levels in neurosyphilis patients. **(A)** ROC curve of CSF CXCL8 in the neurosyphilis and syphilis groups. **(B)** ROC curve of CSF TP in the neurosyphilis and syphilis groups. **(C)** ROC curve of CSF CXCL8 and CSF TP in the neurosyphilis and syphilis groups.Asymptomatic neurosyphilis (n = 28), symptomatic neurosyphilis (n = 54).

Receiver operating characteristic (ROC) curve analysis was utilized to assess the predictive capability of CSF CXCL8 and CSF TP in determining the occurrence of neurosyphilis in syphilis patients. The area under the curve (AUC) for CSF CXCL8 was 0.766 (95% CI: 0.684–0.849, p < 0.001), while for CSF TP, the AUC was 0.830 (95% CI: 0.759–0.900, p < 0.001). The predictive model constructed with these variables yielded an AUC of 0.865 (95% CI: 0.802–0.928, p < 0.001), demonstrating a significantly higher AUC for the combination diagnostics (**Table 3**, **Figure 3**). These results indicate that combined detection enhances diagnostic capability, improving differentiation between patients with neurosyphilis and those with syphilis. Due to the near complete separation observed between CSF RPR results and other indicators during multivariate logistic regression analysis, reliable estimation of predictive probabilities was not achievable; therefore, CSF RPR results were excluded from the ROC analysis.

**Table 3:** Diagnosing value of CSF CXCL8 and CSF TP in patients with neurosyphilis.

Variable	AUC	Standard error	*P*-value	95%(CL)
CSF CXCL8	0.766	0.042	0.000	0.684~0.849
CSF TP	0.830	0.036	0.000	0.759~0.900
Combinations	0.865	0.032	0.000	0.802~0.928

### 3.5 Differences in CSF CXCL1, CXCL5, and CXCL8 levels of neurosyphilis patients before and after treatment

**Figure 4:**
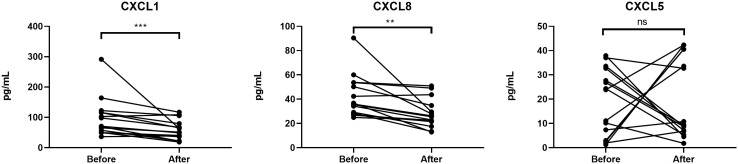
Levels of CSF CXCL1, CXCL8, and CXCL5 of neurosyphilis patients before and after treatment. Data are presented as medians with IQR. Statistical analysis was performed using Wilcoxon signed-rank test. Neurosyphilis patients before and after treatment (n=15). **p < 0.01, ***p < 0.001.

Due to ethical concerns regarding the continuous CSF testing in neurosyphilis patients who clinically exhibit disease remission, we did not perform serial assessments of CSF chemokines in all patients. Ultimately, a follow-up study was conducted on 15 neurosyphilis patients, revealing significant reductions in CSF CXCL1 and CXCL8 levels following standard antibiotic treatment. No significant differences were observed in CSF CXCL5 levels before and after treatment (**Figure 4**). These findings suggest that CSF CXCL1 and CXCL8 levels may serve as biomarkers for effective antibiotic therapy in neurological syphilis patients.

## 4 Discussion

Neurosyphilis signifies a stage wherein *Treponema pallidum* infiltrates the central nervous system. If treatment is delayed, it can result in serious cardiovascular complications and irreversible neurological damage (1). Thus, early diagnosis and prompt intervention are essential for improving patient outcomes. Increasing evidence highlights the significant roles of chemokines in the physiological and pathological processes of the nervous system, with their levels in CSF reflecting changes in inflammatory status (17–19). This study focused on the concentrations of CSF CXCL1, CXCL5, and CXCL8 of patients with neurosyphilis compared to those with syphilis. The aim was to explore the potential role of these chemokines in neurosyphilis. The results showed significantly elevated levels of CSF CXCL1 and CXCL8 in patients with neurosyphilis, which decreased markedly after standard antibiotic therapy. Furthermore, symptomatic neurosyphilis patients exhibited higher levels of CSF CXCL8 than asymptomatic neurosyphilis patients, suggesting a strong correlation between this chemokine and both disease activity and neuronal damage. Notably, multivariate logistic regression analysis identified CSF TP and CSF CXCL8 as independent predictors of neurosyphilis.

Chemokines are small proteins that facilitate cell recruitment and activation in both homeostatic and inflammatory contexts, promoting leukocyte migration to the central nervous system and thereby intensifying the inflammatory response (17, 20). Research has identified CXCL13 as a promising diagnostic marker for neurosyphilis, with elevated levels of CSF CXCL8, CXCL10, and CXCL13 in potentially serving as biomarkers for the condition (21–23). Notably, CXCL1 and CXCL8 are critical to neutrophil recruitment and activation, playing essential roles in inflammatory responses within the central nervous system (24, 25). Their levels are markedly increased in bacterial meningitis as well (26). Additionally, as a potent neutrophil chemoattractant, CXCL8 significantly contributes to the responses to infection and tissue damage (11, 27). Elevated CXCL8 levels have been reported across various diseases, including pulmonary disorders, renal disease, autoimmune conditions, cardiovascular diseases, neurological disorders, and cancer (20, 28). In patients with neurosyphilis, high expression of CSF CXCL1 and CXCL8 likely indicates an intensified intrathecal inflammatory state, which may lead to blood-brain barrier disruption and neuronal injury. Importantly, this inflammatory milieu—rather than merely the *Treponema pallidum* infection—may be the primary driver of neurological symptoms. Notably, the levels of both chemokines significantly decreased following treatment, suggesting a dynamic regulatory mechanism. We propose that CSF CXCL1 and CXCL8 may serve as valuable indicators for assessing the effectiveness of neurosyphilis therapy. Given the current absence of reliable metrics for evaluating intrathecal inflammation and treatment response, these chemokines could enhance clinical assessment. Lastly, CXCL5, a well-established and potent neutrophil chemoattractant, has been shown to play a crucial role in neutrophil recruitment and immune activation in inflammatory conditions such as ulcerative colitis and gout, thereby contributing to the initiation of acute inflammatory responses (26, 29, 30). In the present study, CXCL5 levels did not differ significantly between patients with neurosyphilis and those with syphilis without neurological involvement. However, asymptomatic neurosyphilis patients exhibited elevated CSF CXCL5 levels compared with symptomatic individuals, suggesting that this chemokine may be more prominently involved in the early stages of the disease or in cases characterized by mild neurological impairment.

Additionally, CSF TP serves as a crucial diagnostic and evaluative tool for neurosyphilis. An elevated CSF TP level typically indicates increased blood-brain barrier permeability and heightened intrathecal immune activity (8, 31, 32). This study systematically assessed the predictive value of CSF CXCL8 and CSF TP for diagnosing neurosyphilis using ROC analysis and the AUC. The results demonstrated that both CSF CXCL8 and CSF TP exhibited moderate diagnostic efficacy for neurosyphilis, with AUC values of 0.766 and 0.830, respectively. Notably, the predictive performance improved with the combined application of these two biomarkers, resulting in an increased AUC of 0.865, suggesting that their collaborative use is diagnostically valuable. Moreover, during the construction of the regression model, CSF RPR antibody detection was excluded to avoid issues related to complete separation, thereby allowing for a more robust estimation of other relevant factors. Overall, the combined detection of CSF CXCL8 and CSF TP enhances the early recognition of neurosyphilis. This supports the clinical significance of CSF CXCL8 as a potential biomarker and underscores the complementary role of CSF TP in improving diagnostic accuracy. It further highlights the importance of employing a multi-index comprehensive strategy in diagnosing neurosyphilis. Therefore, further validation of its application value in routine clinical diagnosis through larger sample sizes and prospective studies remains essential.

This study revealed that the counts of CD4+ T cells and B cells in the peripheral blood of neurosyphilis patients were significantly lower than those in syphilis patients. CD4+ T lymphocytes play a critical role in combating the immune response to *Treponema pallidum*. The reduction in CD4+ T cell numbers may indicate a compromised immune surveillance function, thereby facilitating syphilis invasion of the central nervous system. Moreover, sustained antigen stimulation in neurosyphilis patients can lead to immune system depletion and a further decline in lymphocyte counts. Research indicates that the host immune response is pivotal in the pathogenesis of neurosyphilis, primarily manifested as T cell-mediated delayed-type hypersensitivity (2, 32). An imbalance in T cell subsets can diminish cellular immunity, which is reflected in decreased CD4+ T cell levels (33). CD4+ T cells are essential effector cells that activate macrophages, drive the immune and inflammatory responses within the central nervous system, and subsequently contribute to neural damage (34), directly impacting the host’s ability to resist *Treponema pallidum*. The incidence of symptomatic neurosyphilis is negatively correlated with increased CD4+ T lymphocyte counts in the blood (12, 13). Notably, *Treponema pallidum* may inhibit the proliferation of CD4+ T cells by modulating MetAP2, thereby accelerating disease progression (35). However, it is important to note that lymphocyte subsets were analyzed in peripheral blood rather than CSF. Therefore, the observed decrease in CD4+ T cells in the circulation may not fully reflect the immune status of the central nervous system. The immune response within the local CSF plays a crucial role in the pathogenesis of neurosyphilis. Future studies should compare and analyze immune cell subsets in both peripheral blood and CSF to elucidate the migration mechanisms of immune cells and the processes underlying local inflammation.

This study has several limitations. Firstly, it exclusively involved patients from a single medical center, which may introduce selection bias and limit the generalizability of the findings to the broader population of individuals with syphilis and neurosyphilis. Secondly, due to the small sample size and the exploratory nature of the research, results from the subgroup analyses of asymptomatic versus symptomatic neurosyphilis and the comparisons of patients before and after treatment should be interpreted with caution. Additionally, this study primarily aimed to identify potential biomarkers for the diagnosis of neurosyphilis, rather than those reflecting the severity of the condition, which warrants further investigation. Thirdly, another important limitation of this study is the absence of paired plasma samples. Therefore, although elevated chemokine levels were detected in CSF, we could not definitively determine whether these changes reflected true intrathecal production or were partially influenced by systemic inflammatory responses and blood–brain barrier dysfunction. Future studies incorporating simultaneous CSF and plasma measurements are needed to better characterize CNS-specific immune activation in neurosyphilis. Lastly, there is substantial potential to identify and integrate additional biomarkers within the existing predictive framework. By exploring a broader range of biomarkers, researchers can develop more comprehensive and potentially more accurate diagnostic models. The predictive capacity of these combined biomarkers, particularly their synergistic effects, should be tested in larger cohorts.

In conclusion, this study emphasizes the key role of multiple biomarkers in improving the accuracy of diagnosis of neurosyphilis. In particular, the combined application of CSF CXCL8 and CSF TP provides a valuable tool for clinicians. This promising research direction has laid a solid foundation for further improving and optimizing the diagnosis and treatment strategy.

## Data Availability

The datasets presented in this article are not readily available because the data analyzed in this study is subject to the following licenses/restrictions: the datasets generated during and/or analyzed during the current study are available from the corresponding author on reasonable request. Requests to access the datasets should be directed to xuaifangxxh@163.com.
